# Stability and Degradation of Perovskite Solar Cells in Space Environments: Mechanisms and Protocols

**DOI:** 10.3390/ijms27083459

**Published:** 2026-04-12

**Authors:** Aigerim Akylbayeva, Yerzhan Nussupov, Zhansaya Omarova, Yevgeniy Korshikov, Abdurakhman Aldiyarov, Darkhan Yerezhep

**Affiliations:** 1 Laboratory of Engineering Profile, Satbayev University, Satbayev Str., 22, Almaty 040000, Kazakhstan; 2 Department of Electronics, Telecommunications and Space Technologies, Satbayev University, Satbayev Str., 22, Almaty 040000, Kazakhstan; nussupov.erzhan@gmail.com; 3 Department of Standardization, Certification and Metrology, Satbayev University, Satbayev Str., 22, Almaty 040000, Kazakhstan; omarovazhansaya7@gmail.com; 4 Institute of Experimental and Theoretical Physics (IETP), Al-Farabi Kazakh National University, Al-Farabi Avenue, 71, Almaty 050040, Kazakhstan; e.s.korshikov@physics.kz; 5 Faculty of Physics and Technology, Al-Farabi Kazakh National University, Al-Farabi Avenue, 71, Almaty 050040, Kazakhstan; abdurakhman.aldiyarov@kaznu.kz; 6 Technology Commercialization Center, Almaty Management University, Rozybakiyev Str., 227, Almaty 050060, Kazakhstan

**Keywords:** PSC, space photovoltaics, radiation degradation, stability testing, reliability assessment, ISOS protocols

## Abstract

Perovskite solar cells (PSCs) have quickly achieved certified energy conversion efficiency reaching a certified record of 27.3% for single-junction cells, while having a low mass, thin-film form factor and high specific power, which are attractive for space energy systems. However, their long-term reliability in extraterrestrial environments is not adequately ensured by terrestrial qualification routes, and standardized space-related test protocols remain insufficiently developed. This review critically summarizes the current understanding of the degradation of PSCs under the influence of key environmental factors in space—ionizing and non-ionizing radiation, thermal vacuum exposure and thermal cycling, and ultraviolet radiation AM0, as well as atmospheric oxygen in low orbits. The central task of the work is to develop and justify the need to create specialized PSCs test protocols for space applications, since existing ground standards do not reflect the multifactorial nature and extreme orbital loads. It has been shown that thermal vacuum accelerates ion migration, interphase reactions, and degassing, while AM0 UV and atomic oxygen introduce additional photochemical and oxidative mechanisms of destruction; at the same time, stressors often act synergistically and are not detected by single-factor tests. Next, the limitations of the current IEC and ISOS are discussed and an approach to their expansion is formulated through the ISOS-T-Space and ISOS-LC-Space protocols, which integrate high vacuum, AM0 lighting, extended temperature ranges and controlled particle irradiation. It is concluded that the development and interlaboratory validation of such space-oriented protocols is a key condition for the correct qualification of PSCs and targeted optimization of materials and interfaces to meet the requirements of space energy.

## 1. Introduction

In recent years, perovskite solar cells (PSCs) have become one of the most innovative areas of renewable energy sources [1,2,3]. Due to advances in manufacturing technologies such as inkjet printing [4,5], spray-coating [6,7], the slot-die coating method [8,9], and others, PSCs have successfully transitioned from laboratory devices to large-area modules exceeding hundreds of square centimeters, and experimental perovskite photovoltaic panels have achieved an area of several square meters [10,11]. At the same time, the energy conversion efficiency in PSCs has grown rapidly: certified single-junction efficiencies have reached 27.3%, approaching the performance of monocrystalline silicon cells, while perovskite/silicon tandem cells have achieved a certified record of 35.0%, and all-perovskite tandems have surpassed 30% [12]. Due to their high efficiency at a potentially low cost of production, lightness and flexibility of design, PSCs are considered not only for terrestrial applications, but also as promising technology for space energy [13,14,15,16,17]. Their unique properties, such as high specific power (the ratio of output power to mass), low weight and thickness, and the ability to apply thin films to flexible substrates [18,19,20], make PSE attractive for satellites and spacecraft, where every gram counts.

At the same time, the extreme conditions of space place serious demands on the stability and durability of any solar panels. Satellites and interplanetary vehicles operate in ultravacuum conditions, harsh ionizing radiation (protons, electrons, heavy charged particles, gamma quanta), ultraviolet, plasma, and atomic oxygen fluxes, as well as sudden temperature fluctuations and limited illumination away from the Sun [21,22,23,24]. All these factors can cause degradation of solar cells: radiation leads to damage to the crystal lattice and accumulation of defects, ultraviolet light initiates photochemical decomposition, atomic oxygen in low-Earth orbit corrodes unprotected surfaces, cyclic heating and cooling cause thermomechanical stresses. For silicon and gallium arsenide (III–V) solar cells, many years of experience have been accumulated in resisting these factors, for example, special coatings (glasses) are used to protect against protons and oxygen, and the composition and thickness of the layers are optimized for mission tasks [25,26,27,28,29]. However, for PSCs, which are relatively new technology, resistance to space factors is only under study.

It should be noted that PSCs represent one of several emerging photovoltaic material platforms under active investigation for space and high-altitude applications. A broad class of binary oxide ceramics—including TiO_2_, ZnO, Al_2_O_3_, SiO_2_, CeO_2_, Fe_2_O_3_, and WO_3_—is likewise the subject of intensive and comprehensive research for solar cell applications, both as active components and as functional ancillary layers [30]. These materials offer notable advantages including high chemical and thermal stability, radiation hardness, and compatibility with vacuum deposition processes. However, their typically indirect band gaps and low optical absorption coefficients make them unsuitable as primary absorbers for high-efficiency single-junction devices, and their integration with perovskite active layers introduces additional interface engineering challenges. A comparative overview of the principal photovoltaic material classes currently considered for space deployment—including III–V multijunctions, crystalline silicon, thin-film CdTe/CIGS, halide perovskites, and oxide-based architectures—is provided in Table S1, summarising their specific power, radiation tolerance, thermal stability, technological readiness level, and key limitations. Despite encouraging results, such as the ability of perovskites to partially self-repair after radiation damage due to ion migration [31,32,33], or surprisingly low degradation of perovskite samples [34,35,36,37], standardized methods for testing PSCs for space have not yet been developed.

Accordingly, this review systematizes and critically analyzes the current research results of perovskite solar elements in conditions relevant to space applications. The main factors of the space environment and the mechanisms of their effect on PSCs, including cosmic radiation, as well as the effects of temperature, vacuum, ultraviolet radiation and atomic oxygen, are considered. Understanding the mechanisms of degradation under the influence of these factors is necessary for the development of radiation-resistant and durable PSCs, as well as for the creation of adequate test procedures (for example, what types of radiation to carry out, what temperature cycles to set, etc.). A separate section will be devoted to the analysis of existing international standards and protocols testing individual elements to identify their shortcomings and gaps. The purpose of the review is to identify key limitations, unresolved problems and promising areas of development, as well as to form the basis for the development of standardized testing methods and qualifications of PSCs for space energy systems. The present review focuses on five stressor categories with the most extensive experimental evidence base for PSCs: ionizing and non-ionizing radiation, thermal vacuum and thermal cycling, AM0 ultraviolet irradiation, and atomic oxygen exposure. Several additional space-environment factors fall outside the scope of this work, including micrometeoroid and orbital debris impact, spacecraft plasma charging and electrostatic discharge, molecular contamination from outgassing of adjacent components, and secondary neutron flux from GCR interactions with the lunar regolith. These represent important directions for future research.

## 2. Ionizing and Non-Ionizing Energy Loss Mechanisms

**Figure 1:**
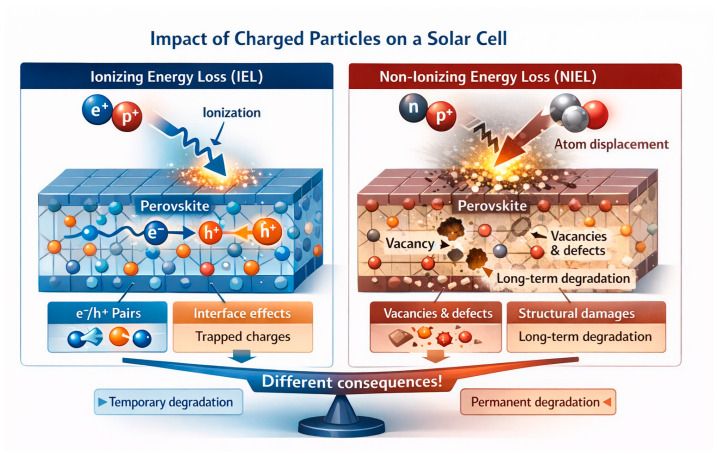
Scheme reflects dominant mechanisms at typical energies; at electron energies above the displacement threshold, both IEL and NIEL operate simultaneously. Original schematic created by the authors based on concepts from [38,39,40].

Charged particles passing through the material of a solar cell transfer energy to it in two main ways: by ionization (excitation of electrons, creation of electron-hole pairs) and by non-ionizing losses (displacement of lattice atoms, creation of vacancies/interstitial defects). These two mechanisms are designated as IEL (Ionizing Energy Loss) and NIEL (Non-Ionizing Energy Loss) respectively [38] (see Figure 1). In the context of radiation resistance, it is important to separate them, since the consequences for the device are fundamentally different.

### 2.1. Ionization Effects (IEL)

High-energy particles (electrons, protons, heavy ions) and electromagnetic radiation (gamma quanta) can knock electrons out of atomic orbitals in a material [41], leading to the formation of free charge carriers, excited states, and—in PSCs—primarily temporary defects including trapped charges, metastable charge states, and local valence changes in the ionic components of the lattice. Due to the ion-soft nature of halide perovskite crystal structures, such ionization-induced defects are largely reversible and can anneal at room temperature, distinguishing these materials from conventional semiconductors [42,43]. Specifically, Herz [42].demonstrated through time-resolved photoluminescence that charge-carrier lifetimes in MAPbI_3_ recover to baseline values within minutes to hours following low-fluence electron irradiation at room temperature, while Azpiroz et al. [43] calculated activation energies of 0.08–0.58 eV for halide vacancy migration, confirming the thermodynamic basis for room-temperature self-annealing. Note: this reversibility has been demonstrated primarily in isolated absorber films; in complete device stacks, transport layer degradation and electrode corrosion introduce additional irreversible channels that reduce the observed recovery [36].

Under purely ionizing conditions—such as gamma irradiation or low-energy electron exposure below the displacement threshold—the crystal lattice remains largely intact, as the transferred energy is insufficient to permanently displace atoms from their lattice sites. However, it is well established that electrons above a material-specific displacement threshold energy (typically 0.1–1 MeV for halide perovskites) can also generate vacancy–interstitial (Frenkel) pairs through elastic nuclear collisions, in addition to ionization effects [44,45,46]. The relative contribution of displacement damage versus ionization depends critically on particle type and energy: irradiation with fast electrons at 1–6 MeV leads predominantly to ionization effects under typical laboratory fluences [14,47,48], but a non-negligible NIEL contribution is present at these energies [44,46]. The dominance of IEL over NIEL therefore reflects relative cross-section magnitudes rather than the complete absence of displacement processes.

IEL-dominated damage causes a moderate decrease in photovoltaic parameters, including short-circuit current density and open-circuit voltage. In silicon and III–V cells, the primary concern is changes in p-n junctions, whereas in perovskites the relevant defects are grain boundary traps and interface states [49]. Although ionization does not destroy the lattice, it can induce chemical changes including radiolysis of organic components and free radical formation [50,51,52,53]. In perovskites, radiation ionization can accelerate the decomposition of MA^+^ or iodide to form PbI_2_, but this pathway typically requires the presence of oxygen or moisture as co-factors [54,55,56,57]. In vacuum or inert atmosphere, these chemical degradation pathways are suppressed, and ionization effects remain limited to electron defect phenomena—charge capture, metastable trap formation, and local valence changes—without leading to significant irreversible phase transformation [58,59,60].

**Figure 2:**
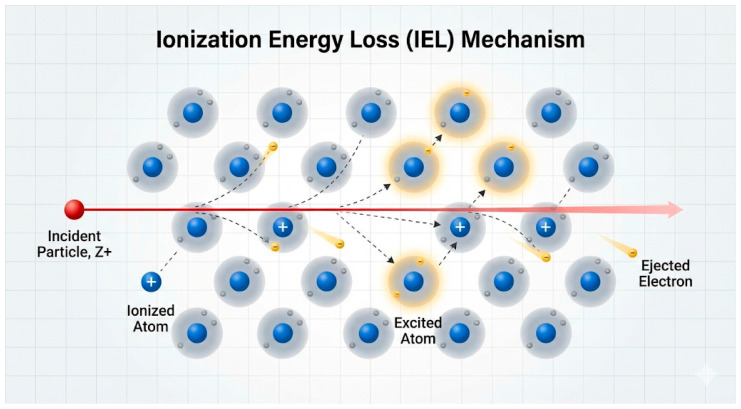
Schematic representation of the mechanism of IEL. Original schematic created by the authors based on [41].

Figure 2 shows a schematic illustration of the passage of a high-energy charged particle through a crystal lattice of a material. The main mechanisms of energy transfer caused by the Coulomb interaction of a particle with the electron shell of atoms are shown, including the processes of ionization, accompanied by the knocking out of electrons, and the excitation of atomic states without loss of an electron. These inelastic interactions are the dominant mechanism of inhibition of charged particles in matter and underlie the formation of ionization damage in PSCs.

### 2.2. Non-Ionizing Effects (NIEL)

This mechanism is relates to how a particle in a collision transfers enough energy to the atoms of the material to displace them from the nodes of the crystal, which forms displacement damage, i.e., vacancies, interstice, complex lattice defects. Non-ionizing losses prevail in collisions of heavy particles (protons, ions) with atomic nuclei [39,40,61]. For each particle–material combination, there is an energy at which NIEL is maximal. In silicon, for example, protons with energies of tens and hundreds of keV cause a strong displacement of atoms, while faster protons (>MeV) travel deeper and spend part of the energy on ionization [62,63,64]. Perovskites are soft crystals with relatively low binding energy, and they are also thin (~300–600 nm layers). For halide perovskites characterized by a small thickness of the absorbing layer, it has been found that low-energy protons with an energy of about 50–150 keV are particularly harmful. This is because in this energy range the contribution of NIEL is maximal and spatially localized within a thin active layer, which leads to the effective formation of atomic displacements and lattice defects [46,65,66]. Modeling has shown that such protons have low penetrating power (on the order of several micrometers in PbI_2_-based materials), and almost all their energy is spent on knocking atoms out of the lattice, creating defects.

As a result, non-ionizing damage generates permanent defects that act as deep recombination centers (nonequilibrium charge carriers recombine non-radiatively) and as traps that reduce current [67,68,69,70]. In halide perovskites, proton NIEL damage in the 50–150 keV range creates predominantly three categories of defects: (a) halogen vacancies (V_I, V_Br)—shallow donors acting as electron traps with capture times on the order of nanoseconds [67]; (b) lead vacancies (V_Pb)—deep acceptors located near mid-gap that serve as efficient non-radiative recombination centers [68]; and (c) interstitial iodine atoms (I_i)—highly mobile species that migrate to grain boundaries and electrode interfaces, accelerating interfacial degradation [66]. Meggiolaro and De Angelis [67] calculated formation energies of 0.1–0.2 eV for V_I defects and capture cross-sections on the order of 10^−14^ cm^2^ using hybrid DFT, establishing their role as shallow electron traps. Alkhalifah et al. [68] demonstrated through non-adiabatic molecular dynamics that V_Pb centres located near mid-gap exhibit non-radiative recombination rates two to three orders of magnitude higher than V_I, providing first-principles quantification of their disproportionate impact on device efficiency. The irreversible reduction of J_sc and FF observed after proton irradiation [71] is attributable primarily to the formation of deep V_Pb recombination centers, whose thermal activation energy (~1.0–1.5 eV) renders them stable at room temperature. The partial recovery of V_oc upon dark storage [31,33], by contrast, reflects annihilation of shallow V_I defects, which have significantly lower activation energies (~0.1–0.3 eV) and recombine spontaneously at room temperature through the ion-soft lattice dynamics characteristic of halide perovskites [42,43,72,73]. These recovery dynamics were characterised in full device stacks [31] and confirmed operando [33]; however, the degree of V_oc recovery is systematically reduced in complete devices relative to absorber films due to competing irreversible degradation at HTL/perovskite and electrode interfaces.

It is important to note that the self-healing behaviour reported across studies is not uniformly reproducible and has been the subject of critical reassessment. Lang et al. [31] reported partial V_oc recovery in n-i-p MAPbI_3_ devices after 68 MeV proton irradiation, attributing this to annihilation of shallow V_I defects. However, Kirmani et al. [36] subsequently demonstrated that this recovery is strongly architecture-dependent: in their systematic comparison, n-i-p devices exhibited partial V_oc recovery but irreversible J_sc and FF losses, while p-i-n devices showed both superior initial tolerance and more complete recovery across all photovoltaic parameters. Critically, Kirmani et al. [32] further showed using dual-energy irradiation that the degree of apparent recovery is strongly influenced by the ratio of IEL to NIEL damage: samples irradiated predominantly via ionisation pathways showed ~60–70% V_oc recovery, whereas those receiving equivalent displacement damage showed <20% recovery. This unresolved dependence on irradiation conditions means that published self-healing claims must be interpreted cautiously, as they may reflect laboratory-specific fluence regimes rather than intrinsic material properties relevant to orbital environments.

This is exactly what was observed in experiments: after a dose of PSE protons, a drop in short-circuit current and fill factor was demonstrated, which did not recover after rest. Thus, NIEL effects are the main factor of long-term degradation. The approach of assessing radiation resistance through NIEL values has long been adopted in space solar cell technology: materials with a lower NIEL coefficient (for a given spectrum of particles) are considered more radiation resistant. In the case of perovskites, we can say that their NIEL behavior is like thin-film semiconductors: they are sensitive to atomic displacements, but less susceptible to charge accumulation than silicon [74].

**Figure 3:**
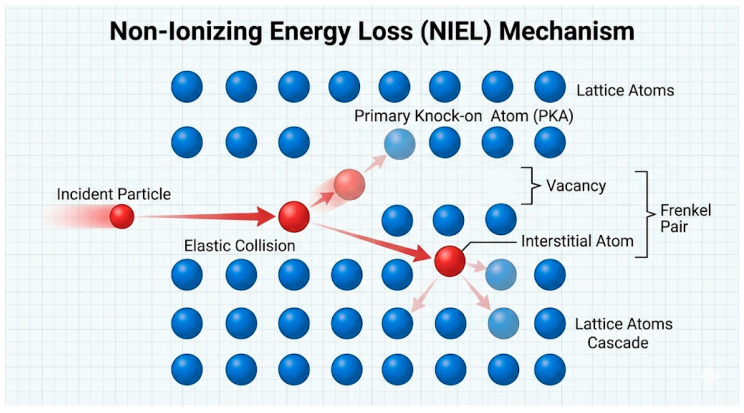
Schematic representation of the mechanism of NIEL and the formation of radiation defects in a crystal lattice. Original schematic created by the authors based on [39,61].

Figure 3 shows a schematic illustration of the process of elastic collision of a high-energy particle with an atom of a crystal lattice. As a result of kinetic energy transfer, the atom is displaced from its node, forming a vacancy and an interstitial atom (Frenkel pair). A primary dislodged atom can initiate a cascade of subsequent displacements, leading to the formation of stable radiation-induced defects in the material structure. Such defects serve as centers of nonradiative recombination and charge traps, which are directly related to the irreversible deterioration of the photovoltaic characteristics of PSCs.

## 3. Mechanisms of Degradation of PSCs in Space

The mechanisms of degradation of PSCs in space largely depend on the orbital radiation environment, which determines the type, energy, and flux of incident particles. Different orbits are characterized by different radiation spectra, leading to qualitatively different dominant damage processes, including ionization, displacement damage, and mixed mechanisms [75,76]. In space, the spectrum of charged particles varies significantly depending on orbital altitude and inclination. Low Earth orbit (LEO, <1000 km) is characterized by a combination of trapped belt particles (protons and electrons), solar energetic particles, and galactic cosmic rays, with the contribution of each component varying with orbital parameters and solar activity [77,78]. Electrons with energies ranging from hundreds of keV to several MeV are mainly concentrated in the outer radiation belt (~5000–20,000 km) but can periodically penetrate LEO [79]. Protons with energies of approximately 50–200 MeV are associated with the inner radiation belt at altitudes of ~1000–5000 km, with peak flux near 1500 km at the geomagnetic equator [80]. In geostationary orbit, spacecraft are continuously exposed to the outer radiation belt, where high-energy electrons of several MeV dominate the background environment [81,82]. Episodic solar proton events can substantially increase proton fluxes at energies of tens of MeV during periods of heightened solar activity, producing short-term but severe radiation stress. Beyond the magnetosphere, the radiation environment is governed by galactic cosmic rays (GCR) and solar energetic particle emissions [83,84]. GCR heavy ions—in particular iron and carbon—cause serious damage through a combination of dense ionization along the particle track and localized atomic displacement [83]. Although the GCR heavy-ion flux is relatively low, its cumulative effect becomes significant over long-duration missions: for deep-space flights exceeding five years, the accumulated GCR dose is among the dominant radiation damage factors [85,86]. On the lunar surface, secondary neutrons produced by interactions of GCR and solar particles with the regolith contribute additional non-ionizing damage through nuclear collisions, accelerating atomic displacement and photovoltaic material degradation [87,88].

Among all space radiation components, low-energy protons in the 50–150 keV range pose the greatest threat to PSC performance. As established in Section 2.2, the NIEL contribution is maximal in this energy range and is spatially concentrated within the thin perovskite absorber layer (~300–600 nm), leading to efficient formation of permanent lattice defects—predominantly halogen vacancies and lead vacancies—that act as deep recombination centers [65,66,71]. Such protons are present in space both as part of the Earth’s radiation belt spectrum and as constituents of solar proton events, making them a pervasive threat across LEO and beyond. For PSCs lacking protective shielding, this impact can lead to significant and irreversible performance degradation. By contrast, electron irradiation leads predominantly to reversible ionization effects: temporary reductions in voltage and fill factor associated with interface charging recover substantially upon dark storage [89,90,91].

A combined analysis of the experimental results and simulation data leads to several conclusions directly relevant to the development of radiation testing protocols for perovskite solar cells intended for space applications. NIEL-based damage assessment—long established as the standard for qualifying silicon and III–V space solar cells—must be systematically extended to perovskite technologies [92], with adaptation for the thin-film absorber geometry, ion-soft lattice, and heightened sensitivity to localized atomic displacements. Qualification strategies relying solely on electron irradiation or total ionizing dose are insufficient to identify the dominant degradation pathways under realistic space conditions, as they neglect the irreversible NIEL contribution from low-energy proton and neutron fluxes. Radiation effects interact synergistically with thermal vacuum cycling, AM0 ultraviolet irradiation, and atomic oxygen exposure; the mechanisms and implications of these interactions are analysed in Section 3.4. The following sections discuss each environmental factor in turn.

### 3.1. Thermal Vacuum Conditions and Thermal Cycles

In addition to radiation, the space environment is characterized by ultra-high vacuum and extreme temperature fluctuations, which create serious thermal and mechanical stresses on photovoltaic devices. Repeated transitions between sunlight and a solar eclipse expose solar batteries to large temperature gradients and frequent thermal cycling throughout their service life, which makes thermal vacuum exposure a critical factor for long-term stability. PSCs are multilayer thin-film systems, usually consisting of a flexible or glass substrate, a metal halide perovskite absorber (~0.5 microns), charge transfer layers (~0.1 microns) and thin metal electrodes. The strong discrepancy between the coefficients of thermal expansion and the mechanical properties of these heterogeneous layers makes perovskite devices particularly sensitive to deformations caused by thermal vacuum exposure, fracture of the interface and the formation of defects [93,94,95]. Therefore, the conditions of thermal vacuum and thermal cycling should be clearly considered in the qualification protocols for PSCs intended for use in space.

The multilayer “sandwich architecture” of PSCs consists of materials with markedly different coefficients of thermal expansion. During cyclic temperature fluctuations in vacuum, where heat exchange occurs solely due to radiation, and convective cooling is absent, these inconsistencies lead to significant thermomechanical stresses at the interface of the layers [96]. In situ studies, [97] that encapsulation alone cannot completely suppress the temperature-induced degradation of halide perovskites. Real-time observations show that elevated temperatures can cause structural and chemical instability in the perovskite layer, which leads to reduced performance even in encapsulated devices. These results indicate that thermal instability is an inherent problem of materials, not exclusively environmental, and should be clearly reflected in reliability assessments and qualification protocols for PSCs intended for space applications.

Witteck et al. [98] demonstrated that vacuum lamination at ≈150 °C causes iodide redistribution across the perovskite/ETL interface within 10 min, producing a 15–20% absolute reduction in fill factor that was not observed under equivalent thermal treatment at atmospheric pressure, confirming that conventional sealing alone is not sufficient to prevent thermal destruction under normal processing conditions. The introduction of internal diffusion barriers—including self-organizing monolayers, conformal oxide layers, and Al_2_O_3_ interlayers deposited by ALD—suppressed this redistribution entirely, preserving FF values above 78% after lamination, which underscores the importance of interface engineering for thermal stability in perovskite photovoltaic technology [99,100,101]. Li et al. [102] independently provided direct experimental evidence that ion migration from the perovskite absorber to the silver electrode is the main mechanism of destruction of inverted PSCs under thermal action, quantifying iodide diffusion through the ETL at 85 °C under vacuum and demonstrating that thermally activated migration rates increase by approximately one order of magnitude relative to ambient conditions.

Using elemental depth profiling and morphological analysis, they showed that iodide and MA^+^ ions diffuse through the electron transfer layer and accumulate at the Ag interface upon heating (~85–100 °C), which leads to the formation of AgI and the associated structural rearrangement at grain boundaries. This ion-controlled interphase reaction leads to a disruption of electron extraction and a noticeable loss of device efficiency, which emphasizes the critical role of thermally activated ion diffusion in the thermal instability of perovskite photovoltaic cells [103]. Laboratory thermocyclic tests of PSCs, for example, involving cooling to −40 °C followed by heating to +85 °C, revealed the formation of microcracks and separation of the interface [104]. These observations highlight the intrinsic vulnerability of perovskite device architectures to mechanical failure caused by thermal vacuum [100,105,106].

In real-world orbital conditions, as already mentioned, the temperature range can be wider. For example, in typical low-orbit missions, including those in near-synchronous orbits (~600–800 km), satellites regularly move from direct sunlight to shadow and back, which leads to repeated thermal cycles with sudden temperature changes [107,108]. During one such cycle, the temperature of the panels can rise to +50…+60 °C in the illuminated area of the orbit and decrease to −20…−30 °C in the shade, which leads to pronounced thermal cycling under conditions of radiative heat exchange. In geostationary orbit, the lighting is constant (except for short seasons of eclipses), and the panels can stably stay about +70…+80 °C under the sun [109]. And on the surface of the Moon, a lunar night cools the panels to ~−170 °C, a lunar day heats them to +120 °C [110,111]. Consequently, the qualification requirements for solar cells inherently depend on the specific mission and must be adapted to the orbital conditions, duration of flight, and prevailing radiation and thermal vacuum effects encountered during operation.

Accordingly, the overall effect of low temperatures on PSCs is manifested in a decrease in their photovoltaic efficiency during extreme cooling. This is due to a combination of physical factors, including a decrease in the mobility and density of free charge carriers due to their partial “freezing”, as well as increased nonradiative recombination through defective states. At lower temperatures, charge traps become energetically deeper and less thermally activated, which leads to an increase in carrier retention time and an increase in the probability of recombination. As a result, there is a decrease in the short-circuit current and a deterioration in charge transfer parameters, especially in devices with a high density of defects and interface states [42,112,113].

Vacuum has a twofold effect on PSCs. On the one hand, the absence of oxygen and moisture eliminates the two most typical external degradation triggers in terrestrial conditions, reducing the likelihood of hydrolysis of the perovskite phase and corrosion processes on the electrodes [99,100]. On the other hand, vacuum promotes desorption and removal of volatile components from the multilayer stack [98]. For hybrid halide perovskites containing organic cations (MA^+^, FA^+^, etc.), heating in vacuum can initiate thermally activated processes of decomposition and desorption of volatile components. These processes are accompanied by the release of gaseous products (for example, CH_3_NH_2_, NH_3_, HI), which leads to a violation of the stoichiometry of the perovskite phase, an increase in the density of defect states and accelerated degradation of interphase boundaries. As a result, the transport properties and stability of interfaces deteriorate, which significantly limits the long-term reliability of devices under thermal vacuum conditions [103,114,115]. Thus, in vacuum, the uncapsulated PSE lost efficiency due to the release of volatile fragments (for example, the release of iodine gas or organic matter), leading to a change in the stoichiometry of the crystal [116,117]. Therefore, one of the tests introduced for space is thermovacuum: holding samples in a vacuum chamber at cyclic temperatures for a long time.

**Figure 4:**
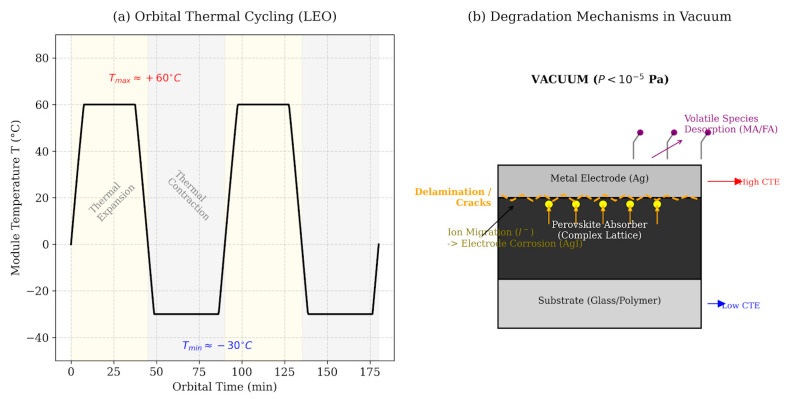
Conditions of orbital thermal cycling and mechanisms of degradation of PSCs in vacuum. Original figure created by the authors based on [98,102,107,110].

Summarizing the experimental and model data described above, it can be concluded that thermal vacuum exposure in the space environment forms a set of interrelated physical and chemical degradation processes affecting both the volume of the perovskite absorber and the interfacial boundaries in the multilayer architecture of the device. In particular, cyclic temperature gradients, the absence of convective heat transfer, and vacuum-induced desorption of volatile components create conditions for the development of thermomechanical stresses, ion migration, and interphase reactions. To illustrate typical orbital temperature profiles and key mechanisms of degradation of PSCs in vacuum, Figure 4 shows a corresponding generalized scheme.

### 3.2. Ultraviolet Radiation and the Solar Spectrum

The solar spectrum outside the Earth’s atmosphere is fundamentally different from terrestrial conditions [118]. There is no atmospheric absorption in space, and photovoltaic devices are exposed to the total solar ultraviolet flux [119]. As a result, space-based solar panels experience a significantly higher photonic energy load than under Earth’s surface (AM1.5) conditions, creating additional photochemical and photophysical stressors that are not amenable to standard ground-based testing. The ultraviolet region accounts for approximately 5–7% of the total solar energy density; however, under terrestrial conditions, a significant fraction of this radiation is effectively shielded by the stratospheric ozone layer, which absorbs photons with wavelengths shorter than ~300 nm almost completely [120]. Consequently, the spectrum reaching the AM1.5 is substantially depleted in the UV relative to the AM0 [121], and the photochemical load on device materials in space is correspondingly higher. For PSCs, this disparity is particularly consequential given the photochemical instability of both the perovskite absorber and the adjacent organic transport layers under high-energy photon exposure.

PSCs based on hybrid halide perovskites contain organic cations—most commonly methylammonium (MA^+^) and formamidinium (FA^+^)—whose susceptibility to UV-induced decomposition represents an intrinsic vulnerability of this material class under AM0 irradiation [122]. In terrestrial conditions, this instability is partially suppressed by atmospheric UV filtration and by the strong above-bandgap absorption of the perovskite layer itself, which converts photons with wavelengths above ~400 nm into photocurrent before they can initiate photochemical reactions.

**Figure 5:**
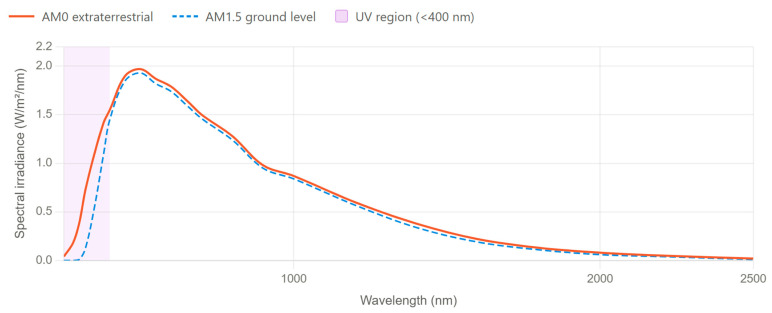
Extraterrestrial solar spectrum (AM0) compared to the ground-level spectrum (AM1.5), with UV sub-regions explicitly indicated (UVC: 100–280 nm, UVB: 280–315 nm, UVA: 315–400 nm). Original figure created by the authors based on [119].

The multilayer architecture of a typical PSC exposes several functionally distinct interfaces to AM0 ultraviolet irradiation, each susceptible to specific photochemical degradation pathways that are largely absent or strongly attenuated under terrestrial AM1.5 conditions (see Figure 5). At the outermost surface, the encapsulation polymer undergoes UV-induced chain scission, yellowing, and progressive embrittlement, which reduce optical transmittance to the underlying active layers and compromise the mechanical integrity of the device stack [123,124,125]. Within the hole transport layer, Spiro-OMeTAD—the most widely used HTL material in conventional n-i-p architectures—is particularly susceptible to photochemical oxidation under sub-400 nm irradiation, leading to charge accumulation at the HTL/perovskite interface, a shift in local energy levels, and a pronounced increase in non-radiative recombination losses [126,127,128]. Inorganic alternatives such as ${NiO}_{x}$ demonstrate significantly improved UV stability under equivalent conditions, which motivates their preferential use in space-qualified device designs, as discussed in Section Device Engineering Strategies for Enhanced UV Stability.

Within the perovskite absorber, two concurrent photodegradation pathways operate under AM0 exposure. First, the MA^+^ cation undergoes photodestruction, yielding volatile CH_3_NH_2_ and HI that desorb from the film under vacuum, disrupting lattice stoichiometry and increasing the density of deep defect states [122,129,130]. Second, photoionization of halogen vacancies ($V_{I}$)—the dominant shallow defect species in halide perovskites—promotes their conversion into deeper trap states, thereby accelerating non-radiative recombination and reducing carrier lifetime [131,132]. At the electron transport layer, UV-induced charge accumulation generates interfacial dipoles that shift energy levels and progressively degrade carrier extraction efficiency [126,127]. These layer-specific mechanisms are largely decoupled under AM1.5 irradiation, where atmospheric filtration eliminates the sub-300 nm photon flux and substantially reduces the UVA/UVB load. Under AM0, however, they act simultaneously and synergistically, producing a cumulative degradation rate that standard terrestrial protocols systematically underestimate. This consideration underscores the mandatory inclusion of AM0-calibrated ultraviolet exposure—covering the full UVC, UVB, and UVA ranges—as an integral component of space qualification protocols for PSCs.

It should be noted that a critical discrepancy in the UV stability literature concerns the role of TiO_2_ as an electron transport layer. Leijtens et al. [127] demonstrated that UV irradiation of TiO_2_-based n-i-p devices leads to rapid, irreversible efficiency loss attributed to photocatalytic decomposition of the hole transport material and generation of deep trap states at the TiO_2_/perovskite interface. In contrast, Pérez-del-Rey et al. [90] reported stable performance of p-i-n devices under simulated space vacuum and electron irradiation, attributing stability to the absence of TiO_2_ in the inverted architecture. These results are not directly contradictory—they reflect different device architectures—but they have been inconsistently cited in the literature as evidence both for and against the UV stability of perovskite devices. The key mechanistic insight is that UV degradation in PSCs is not an intrinsic absorber property but an interface-specific phenomenon whose severity depends critically on the ETL and HTL materials employed. This has a direct implication for testing protocol design: UV stability assessment must be performed on complete device stacks in their final architecture rather than on absorber films, since film-level measurements systematically underestimate degradation in architectures employing TiO_2_ or organic HTL materials.

#### Device Engineering Strategies for Enhanced UV Stability

Several device-level engineering approaches have demonstrated effectiveness in mitigating UV-induced degradation in PSCs, which is directly relevant to space qualification. First, replacement of organic hole transport layers with inorganic alternatives represents one of the most impactful strategies. Devices employing NiO_x_ or CuSCN as hole transport layers exhibit significantly superior UV stability compared to conventional Spiro-OMeTAD-based architectures, as the latter undergoes rapid photochemical oxidation under UV irradiation [128]. In that study, NiO_x_-based inverted PSCs retained over 90% of initial efficiency after extended UV exposure under conditions simulating the UV component of the AM0 spectrum, whereas Spiro-OMeTAD devices lost more than 50% of their performance under equivalent conditions. Second, two-dimensional/three-dimensional (2D/3D) perovskite heterostructures introduced at the absorber surface have been shown to act as UV-stable passivation layers that simultaneously suppress ion migration and reduce photon-induced decomposition of the organic cation sublattice [133]. The 2D capping layer absorbs a portion of the sub-400 nm photon flux before it reaches the bulk 3D absorber, reducing the photochemical load on the MA^+^/FA^+^ cations. Third, A-site cation engineering—specifically the partial or full substitution of MA^+^ with the more thermally and photochemically stable FA^+^ or inorganic cesium (Cs^+^)—reduces the rate of photodecomposition pathways involving volatile organic products [122,129].

### 3.3. Atomic Oxygen in Low Orbit

Atomic oxygen is one of the most chemically aggressive factors of the space environment, characteristic of LEO. In the upper atmosphere, molecular oxygen (O_2_) dissociates under the action of ultraviolet radiation and aerodynamic heating, forming a rarefied flux of highly reactive individual oxygen atoms [134,135,136,137]. At an altitude of approximately 400 km—representative of the International Space Station orbit—under moderate solar activity conditions (solar flux index F10.7 ~ 150), the annual fluence of atomic oxygen is estimated at ~10^20^ atoms·cm^−2^, representing an average scenario. Under solar maximum conditions, the expanded thermosphere increases AtOx density at 400 km by a factor of 2–5, while at higher LEO altitudes (600–800 km) the annual fluence decreases to ~10^18^–10^19^ atoms·cm^−2^. Conversely, at lower altitudes (~300 km) under solar maximum, fluences approaching ~10^21^ atoms·cm^−2^ per year represent the worst-case scenario for unprotected surfaces. Atomic oxygen effectively initiates oxidative erosion of almost all organic materials, including polymers, paint coatings, and organic functional layers, and is capable of degrading thin metal films through accelerated oxidation and associated changes in optical properties [138,139,140,141].

Silicon and III–V solar cells operated in low-Earth orbits are usually equipped with quartz or borosilicate cover glasses, which effectively shield the semiconductor material from direct exposure to atomic oxygen [142,143,144,145]. Such protective layers prevent oxidative erosion of active areas and preserve the optical and electrical characteristics of devices during long-term operation. In the absence of such barrier coatings, the exposed surfaces of solar cells would undergo rapid chemical degradation under, which highlights the critical role of encapsulation and protective barriers in the design of photovoltaic systems for low-orbit space missions.

PSCs, especially in flexible and ultrathin designs, are often designed without a cover glass to minimize mass and increase specific power. In such architectures, the active layers include organic electron and hole transport layers, the perovskite absorber itself (containing organic cations in hybrid systems), as well as upper electrodes made of organic conductors (for example, PEDOT:PSS) or ultrathin metal films. All these components are potentially vulnerable to the effects of atomic oxygen [146]. Recent tests have shown that when exposed to atomic oxygen (AtOx) without a protective barrier, PSCs lose about 40–43% of their efficiency in just 2 h, while devices with a thin oxide coating retain >97% PCE, which indicates the key role of high-quality encapsulation in space conditions [147]. Also, [148] also showed the simultaneous effect of atomic oxygen and ultraviolet radiation, which leads to synergistically enhanced destruction of polymer materials, whereas the developed coating from the metal–organic framework MIL-53(Al) significantly reduces erosion, demonstrating an erosive yield of only ~4.4 × 10^−25^ cm^3^/atom (≈12% of the raw PI) and emphasizing the critical role of protective barriers in space conditions. Accordingly, the development of flexible MOF coatings such as MIL-53(Al) demonstrated a significant increase in resistance to erosion by atomic oxygen: the erosion yield was only ~12% of the value for the untreated polymer, and the coating retained its structural integrity after numerous bending cycles, which makes such barriers promising for protecting photovoltaic materials in conditions of low-orbit environment [149]. The use of modified barrier coatings, such as POSS hybrid composites, significantly increases the resistance of materials to erosion by atomic oxygen, which is manifested in a reduction in wear and weight loss by more than 80% compared with unmodified polyimide coatings [150].

These results collectively establish that atomic oxygen erosion is not a secondary concern for PSC space deployment but a primary failure mechanism for any unprotected organic component. The erosion yield data—ranging from ~10^−24^ cm^3^/atom for unprotected polyimide to ~4.4 × 10^−25^ cm^3^/atom for MIL-53(Al)-coated surfaces [148] and <10^−25^ cm^3^/atom for ALD oxide barriers [147]—provide a quantitative basis for barrier material selection that should be directly integrated into device design specifications prior to space qualification testing.

**Figure 6:**
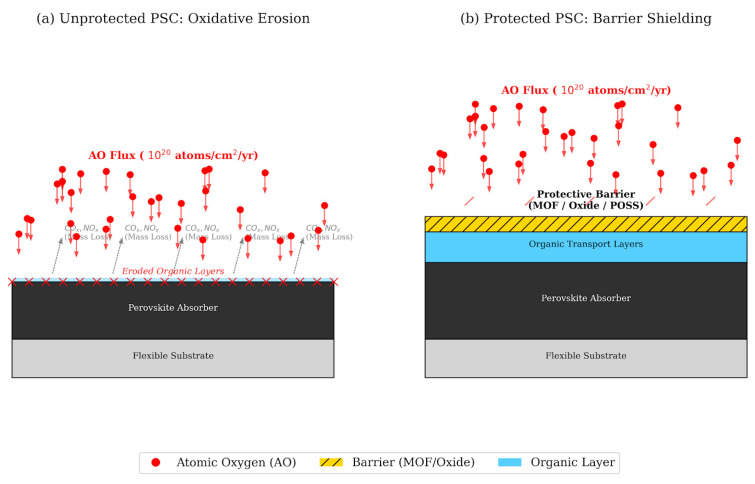
The effect of atomic oxygen on the degradation of PSCs and the role of protective barrier coatings. Original figure created by the authors based on [147,148].

Figure 6 schematically shows the effect of atomic oxygen on the degradation of unprotected PSCs, as well as the effectiveness of various barrier coatings in suppressing oxidative erosion and maintaining the functional characteristics of devices in a low-orbit space environment.

### 3.4. Synthesis of Space Degradation Mechanisms and Current Experimental Evidence Base

The preceding sections have systematically examined the four principal categories of space-environment stressors affecting PSC performance: ionizing and non-ionizing radiation (Section 2), thermal vacuum cycling (Section 3.1), AM0 ultraviolet irradiation (Section 3.2), and atomic oxygen exposure (Section 3.3). Before proceeding to the analysis of existing testing standards and the proposal of space-adapted protocols, it is necessary to synthesize these findings into a coherent mechanistic picture and to critically evaluate the current experimental evidence base against which any qualification framework must be validated.

A key conclusion emerging from Section 2, Section 3.1, Section 3.2 and Section 3.3, is that the degradation of PSCs in space is inherently multifactorial and that the individual mechanisms do not operate independently. Thermal vacuum conditions accelerate ion migration and volatile component desorption, thereby increasing the density of mobile defects available to act as recombination centers [98,102,114]. Superimposed AM0 ultraviolet irradiation photoionizes these pre-existing defect states and initiates photochemical decomposition of organic cation sublattices, compounding the structural disorder introduced by thermal cycling [122,129,130,131,132]. Atomic oxygen in LEO adds an oxidative erosion pathway that is particularly severe for unprotected organic transport layers and thin metal electrodes [147,148]. Ionizing radiation from trapped belt particles and solar energetic events then acts upon a material that has already been structurally and chemically modified by the preceding stressors, producing damage cascades whose magnitude and reversibility differ substantially from those observed in single-stressor laboratory experiments [37,75]. This synergistic interaction between stressors is not captured by any currently standardized testing protocol, as discussed in detail in Section 4.

A second key conclusion concerns the distinction between ionization-dominated and displacement-dominated damage regimes and their differential consequences for device performance and recovery. As established in Section 2, IEL-dominated damage generates largely reversible defects, whereas NIEL-dominated damage from low-energy protons produces permanent lattice vacancies with irreversible efficiency losses [31,71,151].

The coexistence of both damage regimes in real orbital environments, combined with their differential sensitivity to co-stressors, means that qualification strategies based solely on total ionizing dose or electron irradiation systematically underestimate long-term degradation under proton-rich environments.

A third conclusion of practical importance concerns the strong dependence of degradation behavior on device architecture, absorber composition, and encapsulation strategy. The available experimental data indicate that inverted (p-i-n) device configurations consistently exhibit superior radiation tolerance compared to conventional (n-i-p) architectures, that inorganic and mixed-cation absorber compositions outperform MA-rich perovskites under combined UV and thermal stress, and that effective barrier encapsulation is an absolute prerequisite for survival under atomic oxygen exposure [36,147,151]. These architecture-dependent effects must be explicitly accounted for in any qualification protocol intended to produce results that are transferable across different device designs.

To provide a structured and accessible reference for the current experimental evidence base, Table 1 consolidates the key published studies on PSC stability under space-relevant conditions. For each study, the device architecture, absorber composition, encapsulation approach, substrate type, applied test conditions, and principal findings are summarized; the table additionally distinguishes between absorber-film-only measurements and full device stack evaluations, a distinction that is critical for the correct interpretation of degradation data in the context of space qualification, as discussed in Section 2. Direct quantitative comparison across studies is complicated by significant variability in irradiation conditions, measurement protocols, and device architectures: studies reporting PCE retention values were conducted under fluences ranging from ~10^10^ to ~10^14^ p/cm^2^ or e/cm^2^, spanning four orders of magnitude, which precludes straightforward cross-study benchmarking. The table is therefore structured to highlight mechanistic findings and architectural trends rather than to rank absolute radiation tolerance across incompatible experimental conditions.

The data consolidated in Table 1 permit several observations of direct relevance to protocol development. First, with respect to architecture dependence, p-i-n (inverted) devices consistently demonstrate superior radiation tolerance and partial recovery characteristics compared to n-i-p configurations across multiple independent studies [14,32,90,151], which is attributed to lower interfacial defect density and reduced ion migration pathways in the inverted stack [36]. This systematic difference implies that qualification results obtained on one architecture cannot be directly extrapolated to the other, and that space-adapted protocols should specify device architecture as a mandatory reporting parameter.

Second, with respect to the real-orbit evidence base, the only available data from actual orbital exposure [34,35] were obtained for encapsulated devices on rigid substrates operating in LEO aboard the ISS. Flexible device configurations—which offer specific power advantages of one to two orders of magnitude over rigid glass-substrate devices and are therefore strategically most relevant for lightweight space power systems—remain entirely uncharacterized under real orbital conditions. This gap represents the most critical deficiency in the current evidence base and cannot be addressed without either dedicated on-orbit experiments or sufficiently validated ground-based simulation protocols.

Third, with respect to multi-stressor versus single-stressor testing, studies that applied combined radiation and environmental stressors [37,75] consistently reported degradation rates and failure modes not observed under any of the individual stressors applied in isolation. In particular, the combination of proton irradiation with elevated temperature and humidity produced irreversible elemental migration and interfacial delamination that were absent in single-stressor controls [37]. While humidity is not directly relevant to the space vacuum environment, the analogous role of thermally activated ion migration under vacuum conditions [102,114] suggests that combined thermal vacuum and radiation testing will similarly reveal synergistic degradation mechanisms invisible to sequential single-factor protocols. This conclusion provides the primary scientific justification for the integrated multi-stressor design of the ISOS-T-Space and ISOS-LC-Space frameworks proposed in Section 5.

Fourth, with respect to the absorber-film versus full-device distinction, several studies in Table 1 report data from absorber films only or from incompletely assembled stacks [40], which limits their direct applicability to device qualification. As discussed in Section 2, self-healing and reversibility characteristics observed in isolated absorber films frequently do not reproduce in full device stacks, where transport layer degradation, electrode corrosion, and interfacial reactions introduce additional irreversible degradation channels [44,45]. Space qualification protocols must therefore be applied exclusively to complete device stacks in their final encapsulated configuration.

A further unresolved question concerns the transferability of ground-based irradiation results to real orbital conditions. The two available on-orbit datasets [34,35] were both obtained in LEO aboard the ISS, which represents a relatively benign radiation environment due to geomagnetic shielding [77,80]. Neither dataset includes measurements at altitudes above the South Atlantic Anomaly, where proton fluxes are elevated by one to two orders of magnitude [77,80], nor in GEO or deep-space environments where the particle spectrum differs qualitatively from LEO [81,83,84]. Furthermore, both ISS studies [34,35] reported minimal degradation over 10-month exposure periods, which stands in apparent contradiction with ground-based proton irradiation data showing significant PCE losses at fluences corresponding to comparable mission durations [71,75]. This discrepancy has not been satisfactorily resolved: possible explanations include geomagnetic shielding effects, encapsulation attenuation of the proton spectrum, and differences in temperature and vacuum conditions between ISS-mounted and ground-irradiated samples [17,36,77]. Resolving this discrepancy is arguably the most critical open question in the field and represents the primary motivation for the multi-stressor ground-based protocols proposed in Section 5.

#### Recovery Mechanisms and Mitigation Strategies for Radiation-Induced Degradation

Radiation-induced performance losses in PSCs can be partly mitigated through both passive materials engineering and active operational strategies. Dark-storage annealing at room temperature partially recovers IEL-induced shallow trap states (V_I) but does not restore deep V_Pb recombination centres responsible for irreversible J_sc and FF losses (see Section 2.2 for activation energy values) [31,42,67,71]. Light-soaking under moderate illumination has been reported to partially restore photocurrent in some perovskite compositions post-irradiation, attributed to photo-assisted defect annihilation [32,33,49]. At the materials level, A-site cation substitution (FA^+^, Cs^+^ replacing MA^+^) reduces the rate of halide vacancy formation and suppresses volatile decomposition pathways, improving both radiation tolerance and recovery kinetics [14,122,129,151]. 2D/3D perovskite heterostructures provide additional surface passivation that limits interstitial iodine migration to grain boundaries post-irradiation [66,133]. Inverted (p-i-n) architectures consistently outperform conventional (n-i-p) configurations in recovery behaviour (Section 3.4) [36,90,151]. These mitigation strategies should be considered as design inputs when selecting materials for protocols at Levels 2 and 3, and recovery measurements (dark-storage J–V after each irradiation step) should be included as a standard characterisation step in the proposed ISOS-T-Space and ISOS-LC-Space protocols [153,154].

### 3.5. Shielding Strategies for Long-Term Space Operation of PSCs

Shielding represents one of the most direct engineering strategies for mitigating radiation-induced degradation of PSCs in space. DeWitt and Benton proposed a weighted shielding effectiveness figure of merit that accounts for the full trapped-particle and GCR spectrum relevant to a given mission, enabling quantitative comparison of shielding materials across mission profiles [155]. For PSCs, the 50–150 keV proton population responsible for maximum NIEL damage (Section 2.2) is particularly relevant: shielding that attenuates this low-energy population has a disproportionate impact on device lifetime relative to its areal mass cost [46,66,71].

Three categories of shielding are relevant to PSC space deployment. First, passive coverglass—borosilicate, fused silica, or cerium-doped glass used in III–V and silicon space cells—provides both proton attenuation and UV filtration and can be applied to rigid PSC modules, with a 100 µm layer raising the proton transmission threshold to ~3–5 MeV [27,28,29]. Second, thin-film ALD oxide barriers (Al_2_O_3_, TiO_2_) provide simultaneous atomic oxygen protection and modest proton attenuation, with devices retaining >97% PCE after ALD-protected atomic oxygen exposure [147]. Third, advanced flexible composites—including polyimide-POSS hybrids [150], aluminium-matrix composites, and MOF-based coatings such as MIL-53(Al) [148,149]—offer attractive specific-mass and flexibility characteristics compatible with rollable PSC architectures. For flexible devices, for which rigid coverglass is incompatible, thin-film and composite barrier strategies represent the primary radiation mitigation pathway and should be incorporated into device design prior to space qualification testing [24,35,146].

## 4. Modern Approaches to Testing and Verification of Solar Panels

The reliable use of solar panels in various terrestrial and extraterrestrial environments depends crucially on reliable testing and verification techniques that can accurately reproduce real-world operating conditions. Traditional qualification protocols, developed mainly for silicon and III–V photovoltaic technologies, have proven their effectiveness in ensuring long-term operation under conditions of well-defined stress factors [156,157]. However, the emergence of new concepts of photovoltaic technology, in particular thin-film and hybrid technologies such as PSCs, has revealed important limitations of traditional testing systems.

Modern testing and verification strategies increasingly use the principles of failure physics, which combine accelerated stress testing with schemes of multifactorial environmental impact to identify synergetic degradation mechanisms [153,158,159].Such comprehensive verification is especially important for applications in space and at high altitudes, where photovoltaic devices operate far beyond standard ground conditions. Consequently, modern testing paradigms are shifting from single-parameter evaluation to integrated validation systems appropriate for tasks designed to bridge the gap between laboratory testing and operational reliability in real conditions.

### 4.1. IEC Standards for Ground-Based Solar Modules

The International Electrotechnical Commission (IEC) has developed a set of unified standards for the qualification testing and certification of photovoltaic modules, widely used in industry, primarily for silicon solar panels [160,161,162]. Central to this system are the IEC 61215 series of standards [163], which regulate long-term reliability tests, and the IEC 61730 standard [164,165], which defines the requirements for the electrical and mechanical safety of modules.

Thermal Cycling (TC) involves repeated temperature excursions between defined limits without humidity control, assessing thermomechanical reliability of device interconnections and interfaces. Damp Heat (DH) testing targets hydrolytic degradation of encapsulants and absorber materials through prolonged combined temperature and humidity exposure. Thermovacuum (TV) testing—the modality most relevant to space qualification—combines cyclic temperature variation with high vacuum conditions, activating vacuum-specific degradation processes including volatile component desorption, stoichiometry loss, and accelerated ion migration that are absent under atmospheric conditions. The fundamental distinction is that TC and DH model terrestrial stressors with humidity as the dominant degradation driver, whereas TV models orbital conditions where vacuum and temperature dominate. The application of DH testing to space qualification of PSCs is therefore methodologically inappropriate: it eliminates precisely the environmental agent that dominates terrestrially but is absent in orbit, while failing to activate the vacuum-specific processes that dominate in space.

IEC 61215 specifies the following stress tests simulating long-term terrestrial operation. The standard thermal cycle covers −40 °C to +85 °C at 85% relative humidity for 200 cycles, assessing resistance to thermomechanical stresses. The Damp Heat test (DH 85/85) is conducted at 85 °C and 85% relative humidity for 1000 h, evaluating long-term resistance to moisture and temperature. UV irradiation involves a total dose of 15 kWh/m^2^ in the 280–400 nm range at (60 ± 5)°C, equivalent to several years of terrestrial solar UV exposure. Photoinduced aging is assessed at (1000 ± 100) W/m^2^ irradiance; for bifacial modules, the rear side receives (300 ± 30) W/m^2^.

The direct application of these standards to PSCs in space is limited for three reasons [153,166,167]. First, the temperature range of −40 to +85 °C is insufficient for orbital missions: in vacuum, panels cool to −150…−170 °C during eclipse and reach ≈+100 °C or higher under direct solar irradiation [168,169]. Moreover, LEO spacecraft undergo ~15–16 thermal cycles per day (~5840 per year), orders of magnitude more than the ~200 cycles specified by IEC 61215 [170], making the standard inadequate in terms of both cycle count and temperature amplitude. Second, IEC qualification does not include radiation exposure as a mandatory test component, since for terrestrial operation radiation damage is secondary to climatic loads. In space, however, electron and proton irradiation is a primary reliability factor and must be explicitly incorporated into the verification program. Third, while the IEC humidity tests are irrelevant to the orbital vacuum environment, vacuum itself introduces degradation pathways—outgassing, volatile component desorption, and stoichiometry loss—that require dedicated thermovacuum testing not covered by any current IEC procedure.

The inadequacy of IEC 61215 for space qualification can be quantified concretely. The standard specifies 200 thermal cycles over the range −40 to +85 °C, representing a total temperature excursion of 125 °C per cycle [163]. A LEO spacecraft at 600 km altitude experiences ~5435 cycles per year with excursions of up to 90 °C per cycle under radiative-only heat transfer conditions [107,108,171]. The IEC 200-cycle specification therefore corresponds to less than two weeks of LEO equivalent exposure [172]. Moreover, the IEC UV test specifies a total dose of 15 kWh/m^2^ in the 280–400 nm range [166], which is equivalent to approximately 60 h of continuous AM0 exposure at the UV fraction of the solar constant (1361 W/m^2^ × 0.07 UV fraction) [119,120]. This is three to four orders of magnitude below the cumulative UV dose experienced over a 3-year LEO mission (~26,000 h of illumination) [107,168,169]. These quantitative comparisons demonstrate that IEC standards are not merely insufficient for space qualification—they are categorically mismatched in severity by multiple orders of magnitude [153,166,167].

### 4.2. ISOS Stability Protocols for Laboratory Testing

The ISOS (International Summit on Organic Photovoltaic Stability) protocols, originally developed for organic photovoltaic cells [173], are classified by the type of stress applied into five main categories: dark storage tests, open-air tests, light tests, thermal cycling, and light–humidity–temperature cycling. All these categories remain highly relevant for PSCs. Each category includes three levels of complexity—basic, intermediate, and advanced—designed for different levels of laboratory infrastructure. The basic level (Level 1) assumes the use of readily available equipment and limited control of stress factors but is considered the minimum acceptable standard for stability assessment. Levels 2 and 3 require specialized equipment, including environmental chambers and maximum power point tracking systems, and provide higher reproducibility and reliability of results. The protocols are applicable to both sealed and unsealed devices, provided they are clearly specified, including inert atmosphere test conditions.

Accordingly, long-term stability is one of the key factors determining the prospects for the practical application of PSCs. Despite significant progress in achieving high efficiencies, device durability results remain difficult to compare due to differences in experimental conditions, measurement methods, and data presentation formats. In this regard, international ISOS protocols, originally developed for organic solar cells and subsequently adapted for PSCs, are particularly important. Thus, the article proposes a more extensive consensus approach to the use of ISOS protocols considering the specific degradation mechanisms of perovskite materials, including ion migration, metastability, and photoinduced phase transformations [153].

ISOS-L protocols are aimed at studying the degradation of PSCs under continuous illumination. This mode simulates long-term operation of the device under solar radiation and allows for the identification of photoinduced mechanisms of destruction of the active layer and interfaces. Light exposure tests initiate and enhance the migration of ions and defects in the structure of PSCs [154,174,175,176] and promote phase separation in the photoactive layer [51,177], which leads to a progressive decrease in photovoltaic efficiency. Under the influence of illumination, the processes of redistribution of ion vacancies, accumulation of charges at interfaces, and rearrangement of the crystal lattice are activated, accompanied by an increase in recombination losses [178]. It should be noted that when considering continuous illumination, special attention is paid to the characteristics of the light source, including the spectral composition, the presence of an ultraviolet component, and the stability of intensity. Using different lamp types without proper calibration can lead to significant discrepancies in results. To accurately assess efficiency, it is recommended to take hysteresis into account and conduct measurements in a quasi-steady-state mode or using maximum power point tracking algorithms.

In outdoor ISOS-O stability studies, device degradation occurs under the influence of natural solar radiation and realistic atmospheric conditions. Despite the limited reproducibility of such tests due to their dependence on weather factors, geographic location, and seasonal variations, field tests have the highest practical relevance for assessing the operational longevity of photovoltaic devices. In contrast to accelerated laboratory protocols, ISOS-O tests directly provide realistic, albeit climate-sensitive, estimates of solar cell lifetime. An additional advantage of ISOS-O is the ability to use experimental data to determine acceleration factors that relate accelerated test results to lifetime in a natural environment. A similar approach was previously used in the development of the IEC 61215 standard, based on a comparison of field and laboratory data for silicon modules [160,179]. Despite the high importance of this area, studies of the stability of PSCs under outdoor conditions currently remain limited [180,181,182]. However, important results have already been obtained, including the identification of the key role of light-dark cycles in degradation processes [182] and the observation of anomalously high open-circuit voltage values at low light intensities [181]. These observations highlight the unique behavior of PSCs under real-world conditions and the need for further development of protocols.

ISOS-D dark storage tests are aimed at assessing the stability of PSCs when exposed to oxygen, moisture, and other chemically active atmospheric components naturally present in the environment, including CO_2_, NO_x_, and H_2_S, as well as to elevated temperatures. These tests simulate operating conditions under which the photovoltaic device is not exposed to light and allow the identification of degradation processes caused primarily by chemical and thermal instability of the materials. The surrounding atmosphere plays a decisive role in determining the durability of perovskite absorbers and functional transport layers [50,183,184]. Interaction with oxygen and moisture initiates redox reactions, hydrolysis of organic cations, and disordering of the crystal lattice, accompanied by the formation of vacancies, ion defects, and deep charge traps [170,185,186]. Energy barriers form at the interfaces, enhancing nonradiative recombination and leading to gradual degradation of photovoltaic characteristics. Additionally, adsorption of atmospheric molecules on the perovskite surface and transport layers can induce local surface charges, altering the ion distribution and spatial distribution of the electric field within the device. These processes contribute to increased hysteresis in the current-voltage characteristics, increased leakage currents, and reduced operational stability even in the absence of light exposure.

Within the ISOS-D-1 protocols, tests are conducted in a natural laboratory atmosphere with temperature and relative humidity monitoring without their active control [187]. Standard conditions (23 °C) are assumed to be maintained, which ensures a basic level of comparability of results and is considered a minimum requirement for assessing long-term chemical stability. This level allows for the identification of the most vulnerable materials and interfaces at early stages of degradation. More advanced ISOS-D-2 and ISOS-D-3 levels involve the use of climatic chambers with specified temperature and humidity parameters, including accelerated aging modes at elevated temperatures and controlled humidity. Thus, elevated temperatures are widely used to assess the thermal stability of solar cells and to accelerate degradation processes induced by other stress factors [188]. Thermal degradation in the dark in PSCs is caused by chemical and structural instability of the absorber materials and transport layers [189,190], as well as thermally activated interfacial reactions [191]. It should be noted that many metal halide perovskites undergo phase transitions in the temperature range relevant to photovoltaic applications. The impact of these transitions on the durability of devices remains poorly understood, which complicates the interpretation of thermal aging results.

ISOS-T dark thermal cycling and combining light, temperature, and humidity cycling are widely used to evaluate the degradation pathways of photovoltaic devices caused by diurnal and seasonal variations in solar radiation, temperature, and humidity. These protocols allow realistic modeling of outdoor operating conditions and provide critical information on the failure mechanisms associated with interface delamination and contact degradation [100]. Consequently, they have been included in qualification standards for photovoltaic technologies [153,166]. These testing approaches are particularly relevant for PSCs, which exhibit pronounced sensitivity to thermomechanical and ion migration-induced stresses. Several studies have shown that temperature cycling causes more severe degradation than constant thermal stress, primarily due to enhanced accumulation and migration of ions at the charge transfer interface under nonequilibrium conditions [192].

While a comprehensive description of all ISOS protocols is available in the foundational consensus statement [153], the present section focuses exclusively on those protocols that carry direct relevance to the stability assessment of PSCs under space-relevant conditions. As summarized in Table 2, protocol categories were evaluated for their applicability to space qualification, based on three criteria: (i) the nature of the primary stress factor and its correspondence to orbital environment stressors, (ii) the temperature range and its adequacy for LEO, GEO, or lunar mission scenarios, and (iii) the test atmosphere and its compatibility with vacuum-induced degradation mechanisms.

Among the existing protocols, ISOS-LT and ISOS-LC represent the most relevant foundations for space-adapted testing, as they combine simultaneous illumination and thermal variation—conditions that most closely approximate the orbital day/night cycle experienced by photovoltaic systems in LEO. The ISOS-T-3 protocol, with its thermal cycling range of −40 °C to +85 °C, provides a useful mechanical screening baseline, but is conducted at ambient pressure and therefore cannot capture vacuum-specific degradation processes such as volatile component desorption and accelerated ion migration. ISOS-L and ISOS-D protocols retain partial relevance for assessing photostability of the absorber layer and thermal aging during eclipse phases, respectively, but require replacement of the AM1.5 light source with an AM0-calibrated simulator and substitution of humid atmosphere with high vacuum to become representative of extraterrestrial conditions. ISOS-O outdoor testing and ISOS-V bias stability protocols are of limited applicability to space qualification and are therefore excluded from the proposed extensions.

Critically, all existing ISOS protocols share a fundamental limitation for space use: they were developed for terrestrial operating conditions and do not account for ionizing radiation, cryogenic temperatures, or the synergistic interaction between simultaneous stressors characteristic of orbital environments. The extension of ISOS-LC and ISOS-T protocols to incorporate these factors forms the basis of the ISOS-LC-Space and ISOS-T-Space frameworks proposed in Section 5 of this review.

A structured overview of the space relevance of existing ISOS protocols and their specific limitations for extraterrestrial qualification is presented in Table 2. For full protocol specifications, including light source parameters, humidity control, and load conditions, the reader is referred to [153].

## 5. Discussion and Proposed Additions to the ISOS-T and ISOS-LC Protocols for Space Applications

While existing ISOS protocols provide a robust and standardized framework (described above) for assessing the stability of PSCs under terrestrial operating conditions, they remain insufficient to replicate the extreme and multifactorial environmental conditions encountered in space applications. Specifically, traditional ISOS procedures do not adequately account for the combined effects of high vacuum, ionizing radiation, cryogenic temperatures, and high-amplitude thermal cycling, which critically impact device degradation in orbital and deep-space environments.

The critical importance of combined—rather than sequential—application of light and thermal stress has been recently underscored by [193], which systematically investigated PSC degradation under simultaneous light and thermal cycling. That study demonstrated that the synergistic interaction between thermally activated ion migration and photoinduced charge redistribution creates a positive feedback loop that accelerates interfacial degradation far beyond what is observed under either stressor applied independently. The synergistic mechanism underlying this design has been confirmed experimentally [193], as discussed in Section 3.4.

To address these limitations, we propose developing enhanced testing protocols, designated ISOS-T-Space and ISOS-LC-Space, specifically designed to simulate realistic space conditions. These protocols are designed to address key environmental factors, including ultra-high vacuum, wide temperature gradients transitioning to cryogenic conditions, variable solar radiation levels, and controlled exposure to high-energy particles and radiation. By integrating these factors into a single testing system, the proposed protocols are intended to provide a more representative assessment of the long-term reliability of devices in space.

Current ISOS-T protocols involve temperature cycling over a range of −40 °C to 85 °C at or near atmospheric pressure. While these conditions are suitable for ground-based qualification, they do not reflect the scale and complexity of temperature variations encountered in spaceflight. In contrast, the proposed ISOS-T-Space protocol extends thermal cycling to a wider temperature range, including cryogenic conditions, while simultaneously operating in high or ultra-high vacuum and controlled radiation fields. This approach enables the investigation of interrelated degradation mechanisms such as radiation-enhanced ion migration, vacuum-induced interfacial instability, and the development of thermally activated defects.

Similarly, the ISOS-LC-Space protocol extends the capabilities of conventional light-dark cyclic tests to include variable illumination profiles reflecting orbital shadowing, eclipse periods, and distance-dependent solar flux variations. Combined with vacuum and radiation forcing, this protocol facilitates realistic modeling of daily and orbital loads in the space environment.

Table 3 summarizes the key differences between traditional ISOS-T procedures and the proposed ISOS-T-Space extensions, which highlight expanded parameter ranges and additional loading factors. Implementation of these advanced protocols is expected to significantly improve the predictive capabilities of laboratory stability assessments and facilitate the rational design of PSCs for next-generation space power systems.

Conventional ISOS-LC protocols evaluate PSC stability under alternating illumination and darkness in ambient atmosphere with AM1.5 irradiation, providing valuable data on light-induced ion migration, phase segregation, and photoinduced degradation under terrestrial conditions [153]. However, two fundamental limitations prevent their direct application to space qualification. First, the AM1.5 spectrum systematically underestimates the photochemical load on organic components and interfaces by ~30–40% relative to AM0, as the terrestrial atmosphere filters the sub-400 nm photon flux that is most damaging to MA^+^/FA^+^ cations and transport layers [119,120,121]. Second, light–dark cycling in ambient atmosphere cannot reproduce the vacuum-specific processes—volatile component desorption, stoichiometry loss, and accelerated ion migration—that are activated during the orbital day/night transition. Recent experimental evidence demonstrates that the simultaneous application of light and thermal cycling produces irreversible interfacial degradation at rates 3–4 times higher than the sum of individually applied stressors [193], confirming that sequential single-factor testing systematically underestimates space degradation rates.

The ISOS-LC-Space protocol proposed here extends the ISOS-LC framework to extraterrestrial conditions by integrating three modifications: replacement of AM1.5 with AM0-calibrated illumination, conduction of light–dark cycling under high vacuum with an orbital period of ~90 min representative of LEO, and incorporation of controlled particle irradiation applied simultaneously with the illumination cycle. This combination enables the investigation of synergistic photochemical, thermal, and radiation-induced degradation mechanisms that are inaccessible to any single-stressor protocol. The key parameters and operating ranges of the proposed ISOS-LC-Space protocol at each qualification level are specified in Table 4.

The three-level structure of both protocols follows the tiered complexity model of the original ISOS framework [153], enabling laboratories with different infrastructure to participate in comparative studies. Level 1 requires standard thermal chambers and inert gas atmosphere and is accessible to most photovoltaic research laboratories. Level 2 requires a vacuum chamber and a particle irradiation source, and is suitable for dedicated space qualification facilities. Level 3 requires ultra-high vacuum, AM0-calibrated solar simulation, and a controlled multi-stressor irradiation setup, corresponding to specialised space environment simulation centres such as ESTEC (ESA) or NASA Glenn Research Center. The fluence values at each level are derived from NIEL-equivalent displacement damage dose methodology applied to thin-film perovskite absorbers [194], based on AP9/AE9 radiation belt models [77]: the 10^11^ p/cm^2^ level constitutes a minimum screening standard accessible to most accelerator facilities, while the 10^12^–10^13^ p/cm^2^ range is recommended for full qualification of devices intended for 3–5 year LEO missions. For mission-specific qualification, fluences should be recalculated using SRIM/TRIM particle transport codes to determine the effective spectrum reaching the perovskite layer after transmission through the device shielding stack. Electrical characterisation at all levels should include hysteresis-corrected J–V curves measured under standard AM0 (or AM1.5 for Level 1) conditions immediately before and after each stress stage, with stabilised PCE determined by MPP tracking for a minimum of 5 min prior to measurement.

The proton energy range of 50–150 keV specified in the protocol tables corresponds to the energy window in which the NIEL contribution is maximised in PbI_2_-based perovskite absorbers of 300–600 nm thickness, as established through SRIM/TRIM simulations and experimental data [46,151]. The 0.5–1 MeV electron range reflects the dominant outer radiation belt population with demonstrated IEL effects in perovskite devices [14]. Shielding significantly modifies the effective spectrum reaching the absorber: a 100 µm borosilicate coverglass attenuates protons below ~3–5 MeV, eliminating the 50–150 keV population entirely for rigidly encapsulated devices and shifting the dominant damage mechanism toward higher-energy solar proton events. For flexible devices with thin polymeric encapsulants (50–200 µm), the transmission threshold is approximately 1–2 MeV. Users should therefore calculate the device-specific transmitted spectrum using SRIM/TRIM codes before selecting protocol fluence levels.

## 6. Conclusions and Prospects for Further Research

The efficiency and power density of PSCs have reached levels that make them highly attractive for space photovoltaics. However, their long-term reliability in extraterrestrial environments remains inadequately characterized by existing terrestrial standards. This review has established that degradation in the space environment is determined by a complex interaction of ionizing radiation, handling damage, thermal vacuum cycling, ultraviolet irradiation, and atomic oxygen erosion, none of which are fully addressed by current IEC or ISO standards. It has been established that processes dominated by ionizing energy losses, typically caused by electrons and photons, primarily generate metastable defects and partially reversible performance losses, while non-ionizing energy losses associated with low-energy protons lead to persistent lattice damage and irreversible efficiency reduction. This highlights the limitations of qualification strategies based solely on total ionizing dose or electron irradiation and underscores the need for evaluation to ensure the reliability of space-related PSCs. Furthermore, thermal vacuum conditions and ultraviolet irradiation of AM0 significantly accelerate ion migration, interfacial reactions, and mechanical fatigue, while atomic oxygen is a critical factor leading to failures in lightweight and flexible device architectures. These factors act synergistically and cannot be reliably assessed using single-factor testing. Consequently, laboratory protocols developed for ground-based operation systematically underestimate degradation rates in orbital and deep-space environments. To address these shortcomings, this paper proposes the ISOS-T-Space and ISOS-LC-Space conceptual frameworks, which integrate extended temperature ranges, high vacuum, AM0 illumination, and controlled particle irradiation. These protocols provide a physical basis for more realistic and reproducible qualifications of PSCs intended for deployment in space. Future research should prioritize testing for exposure to associated stressors, irradiation campaigns, and interlaboratory validation studies to establish statistically valid performance criteria. In parallel, materials and interface development should focus on preventing shear damage, thermally activated ion migration, and vacuum-induced outgassing. Systematic implementation and validation of space-adapted ISO protocols represent an important step toward internationally accepted reliability standards and will ultimately determine the feasibility of using PSC technologies for next-generation space power systems.

## Figures and Tables

**Table 1:** Summary of key experimental studies on PSC stability under space-relevant stress conditions.

Architecture	Absorber	Encapsulation	Substrate	Test Conditions	PCE Retention/Key Metric	Reversibility	Sample Type	Reference
n-i-p	MAPbI_3_	None	RG	Protons, 68 MeV, ~10^10^ p/cm^2^	Partial PCE recovery after irradiation; self-healing confirmed	PR	FD	Lang et al. [31]
p-i-n	Triple halide Cl/Br/I	None	RG	Electrons, 1 MeV, ~10^14^ e/cm^2^	>85% PCE retained; superior tolerance vs. binary halide	PR	FD	Afshari et al. [14]
n-i-p	MAPbI_3_	None	RG	Electrons, 1 MeV; protons, 10 MeV	Moderate PCE loss; initial radiation tolerance demonstrated	PR	FD	Miyazawa et al. [152]
n-i-p	MAPbI_3_	None	RG	Protons, 150 keV, ~10^11^ p/cm^2^	Irreversible Jsc and FF loss; structural lattice damage confirmed by XRD	IR	FD	Luo et al. [71]
p-i-n	Wide-bandgap	Yes (POL)	RG	Protons, 50–150 keV	A-site PDAI_2_ stabilization significantly improves proton resilience	IR → reduced	FD	Shim et al. [151]
p-i-n	Mixed halide FA/MA/Cs	None	RG	Dual-energy proton irradiation, energy-tuned dosing	Damage and healing mechanisms resolved; NIEL vs. IEL contributions quantified	PR/IR	FD + AF	Kirmani et al. [32]
p-i-n	Mixed halide	None	RG	In situ proton irradiation, operando characterization	Real-time self-healing dynamics observed under continuous proton flux	PR	FD	Sharma et al. [33]
p-i-n	MAPbI_3_	None	RG	Electrons, high vacuum (10^−5^ Pa)	Stable performance under simulated space vacuum; vacuum does not accelerate electron damage	PR	FD	Pérez-del-Rey et al. [90]
n-i-p	MAPbI_3_	Yes (GL)	RG	ISS, LEO, real orbit, 10 months	Minimal degradation; first extended real-orbit dataset for PSC	PR	FD	Delmas et al. [34]
p-i-n	Mixed cation/halide	Yes (POL)	FLEX (PET)	ISS, LEO, real orbit	First on-orbit evaluation on flexible substrate; moderate PCE retention	PR	FD	Erickson et al. [35]
p-i-n	CsFAPbIBr	Yes (ALD)	RG	AtOx, ~10^20^ atom/cm^2^/yr, LEO-simulated	−40% PCE without barrier; >97% PCE with ALD barrier coating	IR (unprotected)	FD	Seid et al. [147]
p-i-n	Mixed halide	None	RG	Combined: protons + humidity + elevated T	Environmental co-stressors strongly accelerate radiation-induced degradation	IR	FD	Khanal et al. [75]
p-i-n	Mixed cation	None	RG	Protons + humidity + heat, combined exposure	Elemental migration intensified under combined vs. single-stressor conditions	IR	FD	Parashar et al. [37]
p-i-n	Mixed cation	Yes (POL)	FLEX (PEN)	LEO simulation, UV + vacuum	Rollable printed PSC; proof-of-concept for flexible LEO deployment	—	FD	Angmo et al. [24]

Architecture: n-i-p—conventional; p-i-n—inverted. Sample type: FD—full device stack; AF—absorber film only; FD + AF—both reported. Encapsulation: GL—glass–glass laminate; ALD—atomic layer deposition oxide barrier; POL—polymer encapsulant; None—unencapsulated. Substrate: RG—rigid glass; FLEX—flexible polymer (PET/PEN). Reversibility of degradation: PR—partially reversible (20–80% recovery); IR—irreversible (<20% recovery)—not reported.

**Table 2:** ISOS protocols with relevance to space qualification of PSCs and their key limitations for extraterrestrial applications (full protocol specifications available in [153]).

Protocol	Primary Stress	Space Relevance	Key Limitation for Space Qualification
ISOS-T-3	Thermal cycling −40 to +85 °C	Medium	Insufficient T range (vs. −170 to +120 °C in lunar missions); ambient pressure excludes vacuum effects
ISOS-L-2/3	Light soaking at elevated T	Medium	AM1.5 underestimates UV load by ~30–40% vs. AM0; no vacuum; no radiation
ISOS-LC-2/3	Light/dark cycling at elevated T	High	No vacuum; no radiation; AM1.5 spectrum only; basis for proposed ISOS-LC-Space
ISOS-LT-2/3	Simultaneous light + thermal cycling	High	Closest to orbital conditions; still limited by AM1.5 and ambient atmosphere
ISOS-D-2/3	Thermal aging in dark	Medium	Relevant for eclipse/storage phases; humid atmosphere must be replaced with vacuum for space applicability
ISOS-V-2/3	Bias stability in dark	Low–Medium	Relevant for ion migration studies under applied field; not designed for space environmental stressors
ISOS-O	Outdoor natural illumination	Low	Climate-dependent; not reproducible; inapplicable to space environment

**Table 3:** Proposed ISOS-T-Space Protocol.

Parameter	ISOS-T (Current)	ISOS-T-Space Level 1 (Screening)	ISOS-T-Space Level 2 (Qualification)	ISOS-T-Space Level 3 (Full Qualification)	Rationale
Temperature range	−40 °C to +85 °C	−40 °C to +100 °C	−100 °C to +100 °C	−170 °C to +120 °C	Level 3 simulates shadow–sun transitions on lunar surface [110,111] and deep-space missions; Level 2 covers LEO/GEO range [107,108,171]
Heating/cooling rate	~2–10 °C/min	~2–10 °C/min	up to 20 °C/min	up to 30 °C/min (programmable)	Models rapid thermal gradients during orbital day/night transitions
Number of cycles	50–200	≥500	≥1000	≥2000	A spacecraft in LEO at ~600 km altitude has an orbital period of ~96.5 min, yielding ~14.9 thermal cycles per day and ~5435 cycles per year. Level 2 (≥1000 cycles) corresponds to ~67 LEO-equivalent days; Level 3 (≥2000 cycles) to ~134 days. For full mission-life qualification of a 3-year LEO mission (~16,300 total cycles), acceleration factors must be derived by comparing protocol results with available on-orbit data [17,34,172]
Test atmosphere	Ambient/N_2_	N_2_ inert gas	High vacuum (≤10^−4^ Torr)	Ultra-high vacuum (≤10^−5^ Torr)	Mimics space vacuum; activates volatile component desorption and ion migration absent under atmospheric conditions [98,103]
Particle fluence	-	None	Protons 50–150 keV: 10^11^ p/cm^2^ (screening)	Protons 50–150 keV: 10^12^–10^13^ p/cm^2^; electrons 0.5–1 MeV: 10^12^–10^13^ e/cm^2^	Equivalent to 1–5 years LEO proton exposure; based on AP9/AE9 models [77] and NIEL methodology [193,194]
Electrical load condition	OC	OC	Fixed bias at V_MPP	Continuous MPP tracking with hysteresis J–V measurement	OC maximises internal field (conservative/worst-case); MPP tracking is most representative of operational conditions [153,154]
Additional stressors	-	-	-	Coupled radiation + thermal (T + R simultaneous)	Enables detection of synergistic degradation mechanisms invisible to sequential single-stressor protocols [37,75,193]
Sample type	Module/cell	Full encapsulated device	Full encapsulated device	Full encapsulated device in final configuration	Self-healing and reversibility observed in absorber films do not reproduce in full device stacks [36,37]

**Table 4:** Proposed ISOS-LC-Space Protocol.

Parameter	ISOS-T (Current)	ISOS-T-Space Level 1 (Screening)	ISOS-T-Space Level 2 (Qualification)	ISOS-T-Space Level 3 (Full Qualification)	Rationale
Light spectrum	AM1.5	AM1.5	AM0 (extraterrestrial solar spectrum)	AM0 (space solar spectrum)	AM1.5 underestimates UV load by ~30–40% relative to AM0; AM0 mandatory for realistic photochemical stress [119,120,121]
Light intensity	1 sun (1000 W/m^2^)	1 sun	0.5–1.0 sun (adjustable)	0.1–1.5 sun (adjustable)	Simulates solar flux variations across interplanetary distances and orbital shadowing; 0.1 sun models eclipse entry/exit conditions
Light/dark cycle period	2, 8, or 24 h	2 or 8 h	~90 min (LEO orbital period)	~90 min LEO or mission-specific	LEO day/night cycle of ~90 min is the most relevant orbital period for PSC light-cycling degradation [172,193]
Temperature range	up to 85 °C	Ambient	−40 °C to +85 °C	−170 °C to +100 °C	Covers LEO and lunar surface environments; combined light + thermal cycling reveals synergistic degradation not observed under single stressors [193]
Test atmosphere	Ambient air/N_2_	N_2_ inert gas	High vacuum (≤10^−4^ Torr)	Ultra-high vacuum (≤10^−5^ Torr)	Prevents ambient-induced failure mechanisms; activates vacuum-specific desorption and stoichiometry loss [98,103]
Particle fluence	None	None	Protons 50–150 keV: 10^11^ p/cm^2^	Protons 50–150 keV: 10^12^–10^13^ p/cm^2^; electrons 0.5–1 MeV: 10^12^–10^13^ e/cm^2^	Evaluates IEL and NIEL mechanisms simultaneously; fluence based on AP9/AE9 LEO models [77] and NIEL damage methodology [193,194]
Duration	≤1000 h	≤1000 h	≥2000 h	≥3000 h	Long-term AM0 photodegradation studies; 1000 h corresponds to <42 days of continuous AM0 exposure—insufficient for space-grade qualification
Electrical load condition	MPP or OC	OC	Fixed bias at V_MPP	Continuous MPP tracking with hysteresis J–V measurement	Ion migration rate is field-dependent; MPP tracking most representative of operational degradation [153,154,178]
Sample type	Cell/module	Full encapsulated device	Full encapsulated device	Full encapsulated device in final configuration	Space qualification must be performed on complete stacks; film-only results are not transferable to device qualification [36,37]

## Data Availability

The original contributions presented in this study are included in the article. Further inquiries can be directed to the corresponding authors.

## Supplementary Materials

The following supporting information can be downloaded at: https://www.mdpi.com/article/10.3390/ijms27083459/s1.
