# Supplementary material for: Stability and Degradation of Perovskite Solar Cells in Space Environments: Mechanisms and Protocols
Source: Int J Mol Sci. 2026 Apr 12;27(8):3459. doi: 10.3390/ijms27083459 (PMC13116883; doi:10.3390/ijms27083459)
Supplement: Supplementary file 1 [file ijms-27-03459-s001.zip › ijms-4180398-supplementary.pdf]

Table S1. Comparison of photovoltaic material classes for space applications.

Material class	Specific power (W/kg)	Radiation tolerance	Thermal stability	TRL (space)	Key limitations for space
III-V (GaAs, InGaP multijunction)	300–500	High (well-characterised)	Excellent (to ~150 °C)	9 (heritage)	Very high manufacturing cost; heavy coverglass required
Crystalline silicon	~100–200	Moderate	Good	9 (heritage)	Low specific power; brittle; coverglass required
CdTe / CIGS thin film	200–400	Moderate	Moderate	4–6	Cd toxicity (CdTe); In scarcity (CIGS); limited space data
Halide perovskite (PSC)	~1000–3000 (projected)	Promising; partially reversible	Poor for hybrid; improved for inorganic	2–4	Thermal/UV/AO instability; Pb toxicity; no space standards
Binary oxide ceramics (TiO2, ZnO, etc.) as transport/barrier layers	N/A (ancillary)	Generally high	Excellent	5–7 (as coatings)	Not primary absorbers; indirect bandgap limits standalone PV use; integration with perovskite adds interface complexity
